# Astragaloside IV attenuates IL-1β-induced intervertebral disc degeneration through inhibition of the NF-κB pathway

**DOI:** 10.1186/s13018-022-03438-1

**Published:** 2022-12-16

**Authors:** Yueyang Tian, Xu Chu, Qia Huang, Xing Guo, Yuan Xue, Weimin Deng

**Affiliations:** 1 Department of Orthopedics Surgery, Tianjin Medical University General Hospital, Tianjin, China; 2 Tianjin Key Laboratory of Spine and Spinal Cord Injury, Tianjin, China; 3 Department of Immunology, Tianjin Medical University, Tianjin, China; 4 Honghui Hospital, Xi’an Jiaotong University, Xi’an, China

**Keywords:** Astragaloside IV, Intervertebral disc degeneration, NF-κB, Extracellular matrix, Inflammation, Apoptosis

## Abstract

**Background:**

Intervertebral disc degeneration (IDD) is the main cause of low back pain. Patients with low back pain may experience significant socio-economic burdens and decreased productivity. Previous studies have shown that inflammation is one of the main causes of IDD. Astragaloside IV (AS IV), a traditional Chinese medicine, has been reported to have therapeutic effects on many inflammation-related diseases; however, the effectiveness of AS IV as the treatment for IDD has not been studied.

**Methods:**

Nucleus pulposus (NP) cells from patients with IDD were used for the experiments. Cell counting kit 8 (CCK8) was used to evaluate the effect of AS IV on the viability of NP cells (NPCs). To mimic IDD in vitro, NPCs were divided into the following groups: control group, interleukin 1β (IL-1β) group, and AS IV + IL-1β group. To analyse the effect of AS IV on IL-1β-induced IDD, Western blotting, RT-qPCR, flow cytometry, and immunofluorescence assays were performed. To evaluate the effect of AS IV in vivo, a rat model of puncture-induced IDD was established.

**Results:**

AS IV effectively alleviated IL-1β-induced inflammation, apoptosis, and extracellular matrix degeneration in NPCs. We also observed that AS IV decreased the IL-1β-induced phosphorylation of inhibitor of kappa B-alpha (p-IκBα) in the cytosol, and reduced nuclear translocation of NF-κB p65, indicating that AS IV inhibited the NF-κB pathway. Using the puncture-induced rat IDD model, our results showed that AS IV had a protective effect against the progression of IDD, suggesting that AS IV could alleviate IDD in vivo.

**Conclusions:**

Our results demonstrated that AS IV effectively alleviated IDD in vivo and in vitro, indicating that it could be used as a therapeutic to treat IDD.

## Background

Intervertebral disc (IVD) degeneration (IDD) is the main cause of low back pain, which can lead to severe health and economic burdens [1–3]. IVD is located between the vertebral bodies and consists of central nucleus pulposus (NP), surrounded by lamellar annulus fibrosus (AF), and the cartilage endplate [4]. NP is the gelatinous central section of the IVD and is composed of water, proteoglycans (mostly aggrecan), and type II collagen [5]. NP cells (NPCs) are the key cells responsible for maintaining the normal structure and physiological function of IVD. Aberrant physiological behaviour of NPCs, such as higher levels of inflammatory factors, apoptosis, decreased extracellular matrix (ECM) synthesis, and increased ECM degradation, can lead to IDD [6]. Understanding the mechanisms leading to aberrant NPC function is necessary for the targeted treatment of IDD, the subject that has attracted the attention of numerous scientists in this field of research.

Increasing evidence shows that inflammatory responses induced by inflammatory factors are involved in the pathogenesis of IDD [7–9]. Among these inflammatory factors, interleukin 1β (IL-1β) has been widely studied due to its strong pro-inflammatory properties [10–12]. The serum level of IL-1β in healthy people is low; however, the expression of IL-1β is increased in degenerative IVDs, inducing downstream mediators to affect numerous pathological processes involved in IDD [13, 14]. As a result, in NPCs, IL-1β has been implicated in extracellular matrix disorders, inflammation, and apoptosis, all of which are associated with the pathogenesis of IDD [7, 8, 15]. Therefore, the current anti-inflammatory approach has been proved to be an effective treatment of IDD.

Astragaloside IV (AS IV) is the main bioactive ingredient isolated from the traditional Chinese herbal medicine *Astragalus membranaceus* [16] and is reported to have anti-inflammatory [17, 18], anti-oxidant [19, 20], and anti-cancer [21, 22] properties. Previous studies have shown that in osteoarthritis, AS IV can reduce damage caused by inflammation [23, 24]; however, the effect of AS IV treatment on IDD has not yet been investigated.

Here, we demonstrated that the AS IV alleviated IL-1β-induced inflammation, cell apoptosis, and ECM degeneration in human NPCs and investigated possible mechanisms of this effect using both in vitro and in vivo model systems.

## Methods

### NPC culture

This study was approved by the Ethics Committee of Tianjin Medical University General Hospital. NP tissues were collected from 12 patients with IDD (five males and seven females). Human NPCs were isolated as previously described by Risbud et al. [25] The NPCs were maintained in a mixed medium containing DMEM/F12 (Gibco) supplemented with 15% FBS and 1% P/S (Invitrogen) and cultured in a 5% CO_2_ incubator at 37 °C. To maintain cell phenotypes, only the first two passages were used. NPCs were pre-incubated with or without AS IV (MCE, Princeton, NJ, USA, 100 μM) for 2 h, and then treated with 10 ng/mL IL-1β for another 24 h. NPCs treated only with DMEM/F12 medium with 15% FBS containing DMSO were considered as a control.

### Cell viability assay

The effect of AS IV on human NPC viability was measured using a cell counting kit 8 (CCK-8; Dojindo, Tokyo, Japan). Briefly, the cells were seeded into 96-well plates and treated with 0, 10, 20, 50, 100, 200, 500, 1000, and 2000 μM AS IV for 24 h. Next, the cells were incubated with 10  μL of CCK-8 solution, and absorbance at 450 nm was measured using a spectrophotometer (Bio-Rad, California, USA).

### RT-qPCR

After treatments of the human NP cells, RT-qPCR was performed as previously described [26]. *GAPDH* mRNA expression was used to normalize the results. The primers were synthesized by Sangon Biotech (Sangon, Shanghai, China) and are as follows: iNOS(F): 5′‐ACAGGAGGGGTTAAAGCTGC‐3′ and (R): 5′‐TTGTCTCCAAGGGACCAGG‐3′; COX-2(F): 5′‐TCCCTTGGGTGTCAAAGGTAAA‐3′ and (R): 5′‐TGGCCCTCGCTTATGATCTG‐3′; GAPDH(F): 5′‐CCACCCATGGCAAATTCCATGGCA‐3′ and (R): 5′‐TCTAGACGGCAG GTCAGGTCCACC‐3′.

### Western blotting

Cells were lysed using RIPA buffer (Solarbio, Beijing, China) containing protease inhibitors. Nuclear and cytoplasmic proteins from cell lysates were extracted using nuclear extraction reagent (Solarbio), and protein concentration was determined using a BCA kit (Solarbio). Next, proteins were separated by SDS-PAGE, transferred onto PVDF membranes, blocked in 5% non-fat milk, and then incubated with the following primary antibodies: anti-COX-2 (Abcam), anti-iNOS (Proteintech), anti-Bcl-2 (CST), anti-Bax (CST), anti-cleaved-caspase-3 (Abcam), anti-type II collagen (Abcam), anti-aggrecan (Thermo, Rockford, USA), anti-MMP-13 (Thermo), anti-ADAMTS-4 (Abcam), anti-NF-κB p65 (Abcam), anti-IκBα (CST), anti-p-IκBα (CST), anti-GAPDH (Abcam), and anti-histone H3 (Abcam). Finally, the membranes were incubated with corresponding secondary antibodies, and protein bands were detected using an ECL detection system (Millipore). The levels of histone H3 and GAPDH were used as internal controls.

### Immunofluorescence

Immunofluorescence experiments were performed as previously described [27], using antibodies against aggrecan, MMP-13, and p65. DAPI (Beyotime) was used to stain the nucleus.

### Flow cytometry analysis

Cells were digested with trypsin without EDTA (Solarbio), washed with PBS, stained with Annexin V-FITC and PI for 15 min (Keygen, China), and then immediately analysed by flow cytometry.

### Rat IDD model

All animal experiments were approved by the Animal Care and Use Committee of the Tianjin Medical University. SD rats (3-months old) were randomly divided into the IDD group, AS IV + IDD group, and control group. After weighing, 10% chloral hydrate was injected intraperitoneally at a dose of 3.5 mL/kg body weight. To generate IDD, the tail was disinfected, and a 20G needle was inserted from the dorsal to ventral side of the rat caudal disc (Co7/8), rotated 360°, fixed for 30 s, and then removed as described by Han et al. [28]. In the AS IV treatment group (AS IV + IDD), AS IV (50 mg/kg/day) was administered by intragastric injection for 4 weeks. The model (IDD) and sham surgery (control) groups were injected with the same amount of saline.

### Magnetic resonance imaging (MRI)

Eight weeks post-operatively, the animals were anaesthetized as described above and an MRI was performed using the Achieva 3.0T MRI scanner (Philips, USA). The parameters used for T2-weighted imaging were the same as described in the previous study [29]. The degeneration degree was evaluated using the Pfirrmann grading system [30].

### Histological assessment of the rat IDD model

All disc tissues were collected and then processed for haematoxylin and eosin (HE) staining and safranin O-fast green (SO) staining. Images were analysed for the degeneration degree as previously described [31].

#### Statistical analyses

The results are presented as means ± SD of at least three independent experiments, and data were analysed using the SPSS v.25.0 software. Differences between groups were determined using Student’s *t* test or ANOVA followed by Tukey’s test. Nonparametric data (Pfirrmann and histological scores) were analysed using the Kruskal–Wallis test followed by Dunn’s post hoc test. Statistical significance was set at *p* < 0.05.

## Results

### The effect of AS IV on the viability of NP cells

**Fig. 1:**
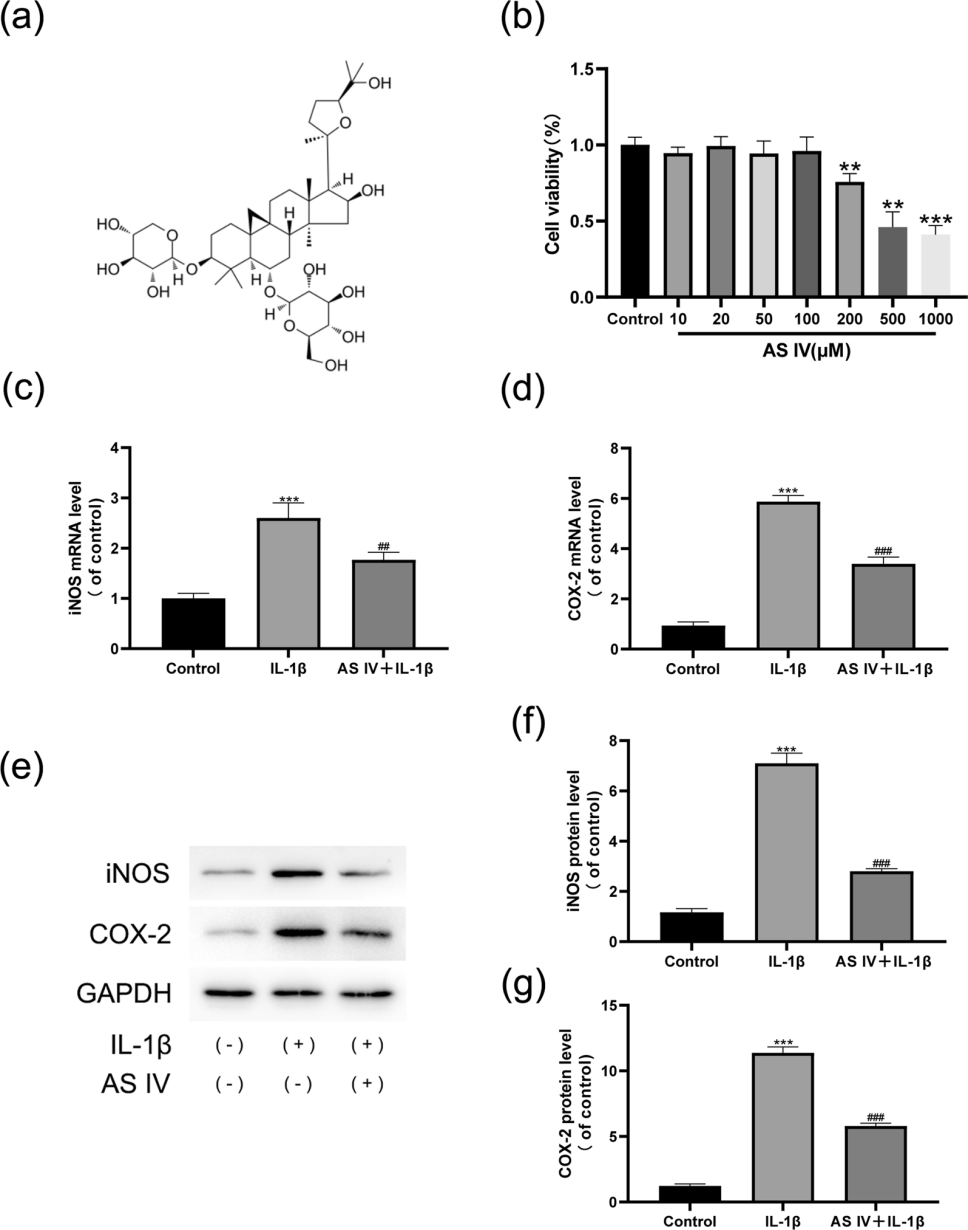
Effect of AS IV on IL-1β-treated NPCs. **a** The molecular structure of AS IV. **b** Cells were cultured for 24 h with AS IV at different concentrations, and cell viability was detected by the CCK8 assay. **c**, **d** iNOS (**c**) and COX-2 (**d**) levels were analysed by RT-qPCR. **e**–**g** COX-2 and iNOS levels were detected using Western blotting. Data are expressed as means ± SD. ***P < 0.001 and **P < 0.01 versus control group. ^###^*P* < 0.001 and ^##^*P* < 0.01 versus IL-1β group

The chemical structure of AS IV is shown in Fig. 1a. To evaluate the effect of different AS IV concentrations on NPC viability, we performed the CCK-8 assay. Our results showed that AS IV concentrations up to 100 μM had no significant effect on the viability of NP cells (Fig. 1b). Therefore, 100-μM concentration was used for subsequent experiments.

### AS IV alleviates IL-1β-induced inflammatory response in NP cells

To evaluate the anti-inflammatory properties of AS IV, we measured the expression levels of iNOS and COX-2, well-established indicators of inflammation, in NPCs cultured under different treatment conditions. The mRNA (Fig. 1c, d) and protein (Fig. 1e–g) levels of iNOS and COX-2 were determined using RT-qPCR and Western blotting, respectively. We observed that AS IV reduced the expression levels of *iNOS* and *COX-2* mRNA and protein induced by IL-1β, suggesting that AS IV effectively alleviated the inflammatory response.

### AS IV alleviates IL-1β-induced apoptosis in NP cells

**Fig. 2:**
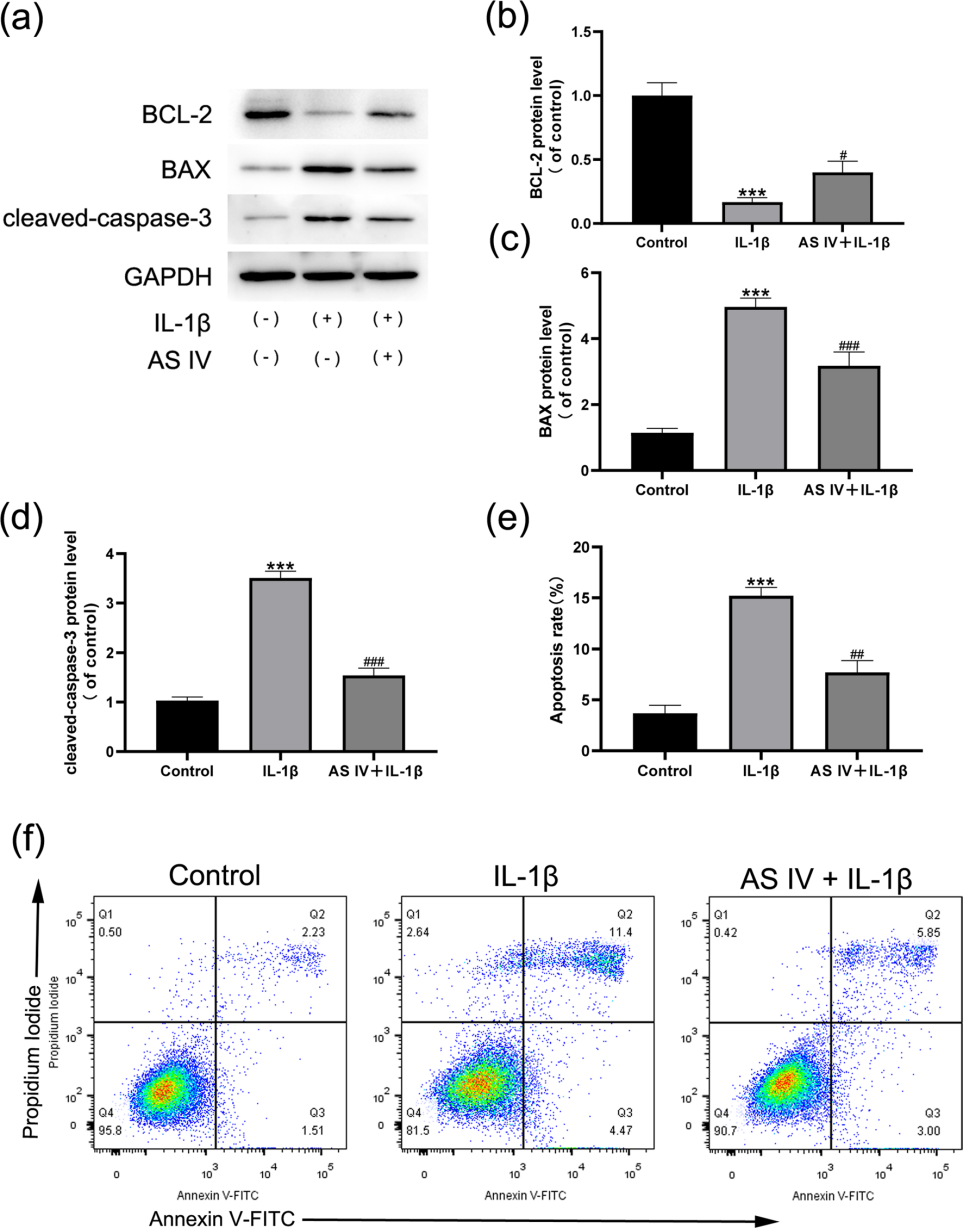
AS IV alleviates apoptosis in NPCs. Cells were incubated with or without AS IV (100 μM) for 2 h, and then treated with 10 ng/ml IL-1β for 24 h. **a**–**d** Cleaved-caspase-3, BAX, and BCL-2 levels were evaluated by Western blotting and then quantified. **e**, **f** Apoptosis in NPCs was analysed by flow cytometry. Data are expressed as means ± SD. ***P < 0.001 versus control group. ^###^*P* < 0.001, ^##^*P* < 0.01 and ^#^*P* < 0.05 versus IL-1β group

To evaluate the effect of AS IV on IL-1β-induced apoptosis, we measured the protein expression levels of apoptosis-related factors. The results of Western blotting showed that IL-1β significantly downregulated the expression of Bcl-2 and upregulated the expression of cleaved caspase-3 and Bax, indicating that AS IV effectively alleviated IL-1β-induced apoptosis in NP cells (Fig. 2a–d). In addition, flow cytometry analysis further confirmed that AS IV treatment significantly reduced IL-1β-induced NP cell apoptosis (Fig. 2e, f).

### AS IV alleviates IL-1β-induced ECM degeneration in NP cells

**Fig. 3:**
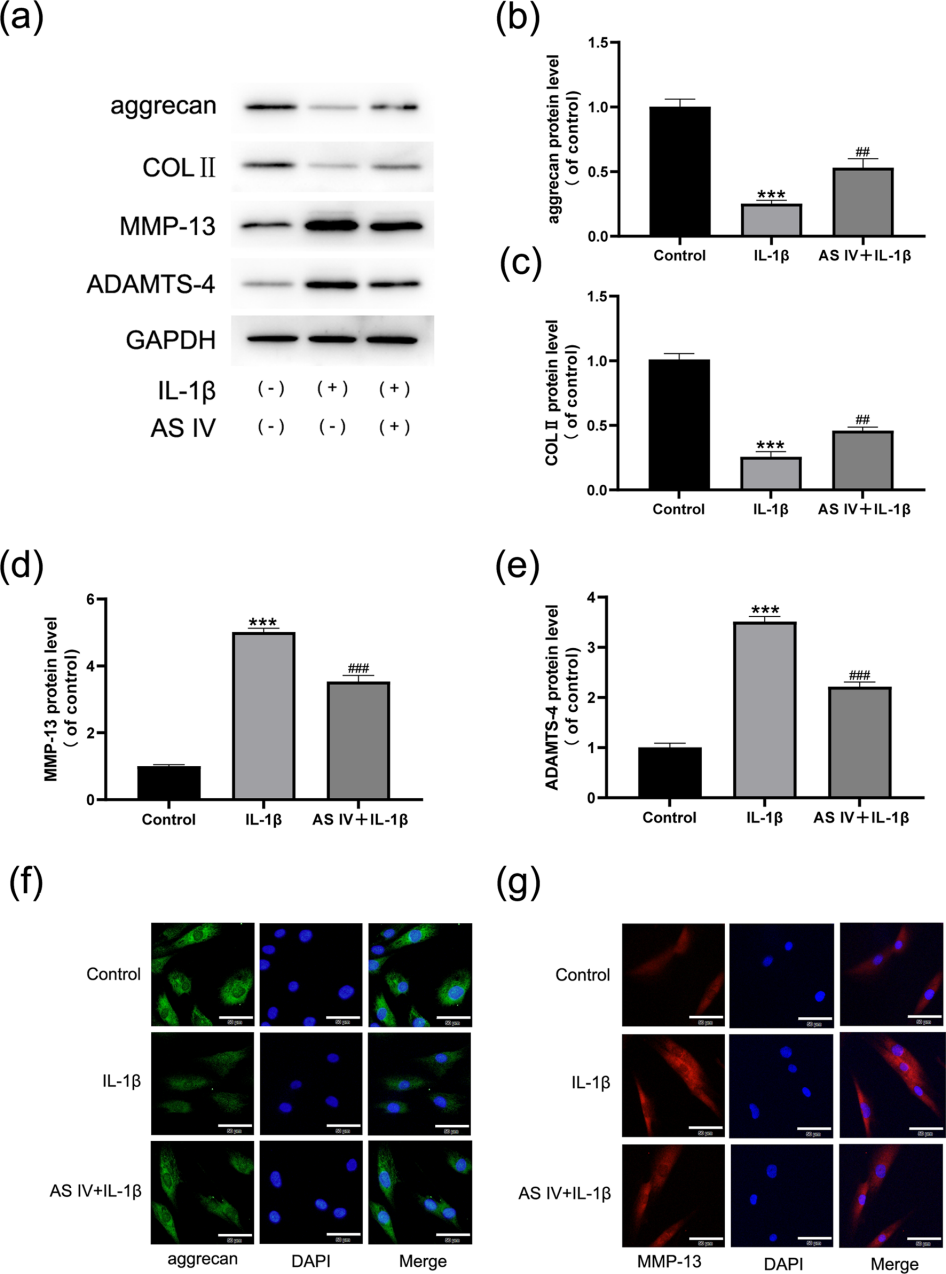
Effect of AS IV on IL-1β-induced NP ECM degeneration. **a**–**e** MMP-13, aggrecan, ADAMTS-4, and COL II expression was evaluated by Western blotting and then quantified. **f**, **g** Immunofluorescence staining of aggrecan (**f**) and MMP-13 (**g**) in the human NPCs. Scale bar: 50 μm. Data are expressed as means ± SD. ***P < 0.001 versus control group. ^###^*P* < 0.001 and ^##^*P* < 0.01 versus IL-1β group

Next, we used western blotting and immunofluorescence to examine the effects of AS IV on the ECM produced by NPCs. IL-1β treatment significantly reduced the expression of type II collagen (Col II) and aggrecan in the NP cells, while the expression of ECM catabolic factors MMP-13 and ADAMTS-4 was significantly increased. AS IV effectively decreased the expression of MMP-13 and ADAMTS-4 and increased the levels of aggrecan and Col II (Fig. 3a–e). Immunofluorescent staining also confirmed that IL-1β-induced aggrecan levels were significantly downregulated and MMP13 protein levels were significantly upregulated. This effect was reversed by AS IV treatment (Fig. 3f, g). These results further indicated that AS IV can effectively alleviate IL-1β-induced ECM degeneration.

### AS IV inhibits the NF-κB pathway activation in NPCs

**Fig. 4:**
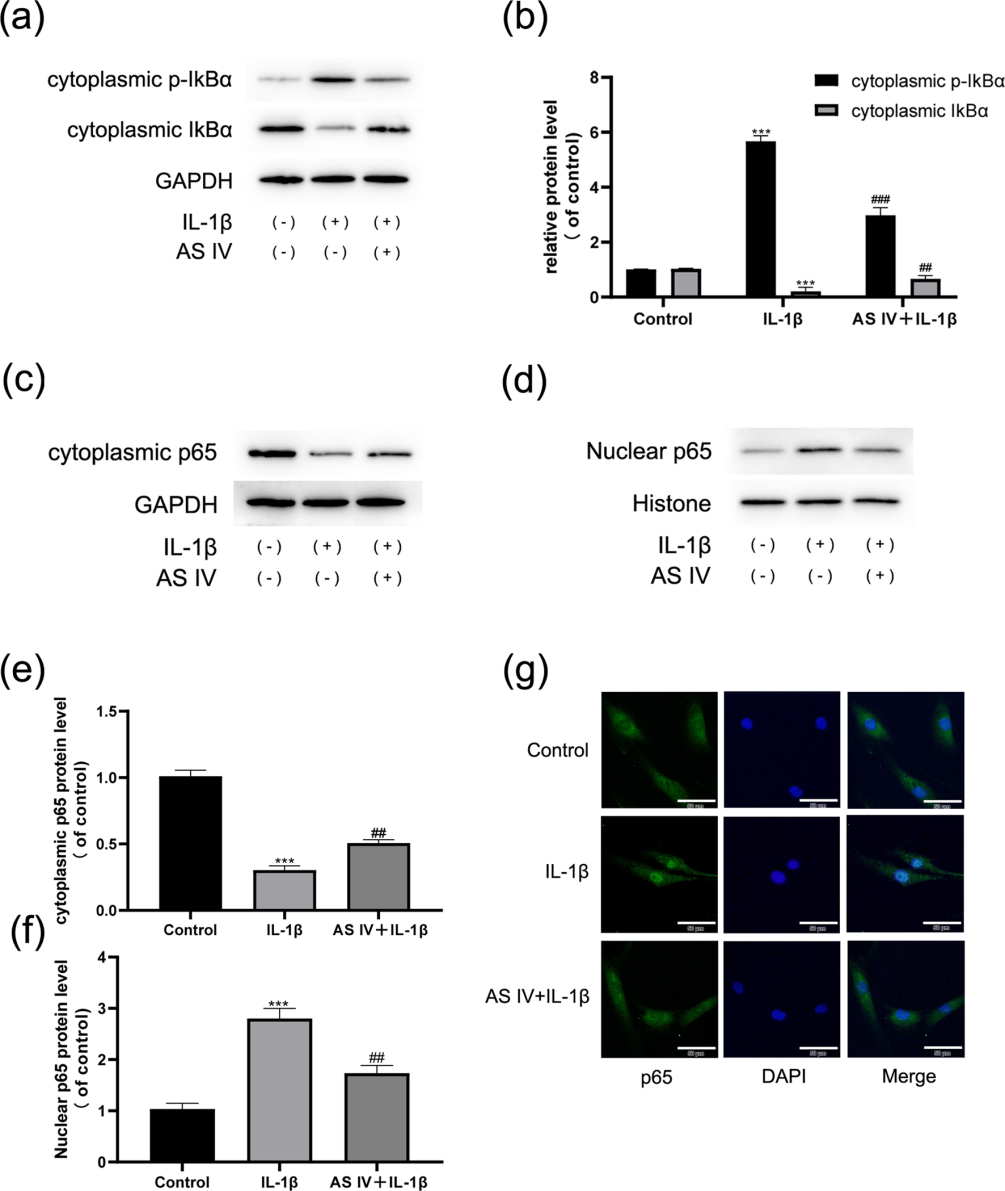
AS IV suppresses NF-κB activation in NPCs. **a**, **b** Cytoplasmic p-IκBα and IκBα protein levels. **c**, **e** Cytoplasmic NF-κB p65 protein levels. **d**, **f** Nuclear NF-κB p65 protein levels. **g** Localization of NF-κB p65 was evaluated by fluorescence microscopy. Scale bar: 50 μm. Data are expressed as means ± SD. ***P < 0.001 versus control group. ^###^*P* < 0.001 and ^##^*P* < 0.01 versus IL-1β group

The NF-κB pathway is one of the key pathways activated by the pro-inflammatory mediators. Therefore, we evaluated the levels of NF-κB pathway-related proteins to further investigate the anti-inflammatory properties of AS IV. The levels of p65, p-IκBα, and IκBα were determined by Western blotting, and our results showed that AS IV significantly reduced p-IκBα levels and the degradation of IκBα induced by IL-1β treatment (Fig. 4a, b). Furthermore, IL-1β significantly decreased cytoplasmic p65 levels and elevated nuclear p65 levels, confirming that IL-1β stimulation significantly increased p65 nuclear translocation. However, this effect was reversed by AS IV treatment (Fig. 4c–f). These findings were further verified using fluorescence microscopy: IL-1β stimulation increased p65 nuclear localization, while AS IV treatment reversed this effect (Fig. 4g). These results showed that AS IV inhibited the IL-1β-induced activation of the NF-κB pathway in NPCs.

### AS IV alleviates puncture-induced IDD in rats

**Fig. 5:**
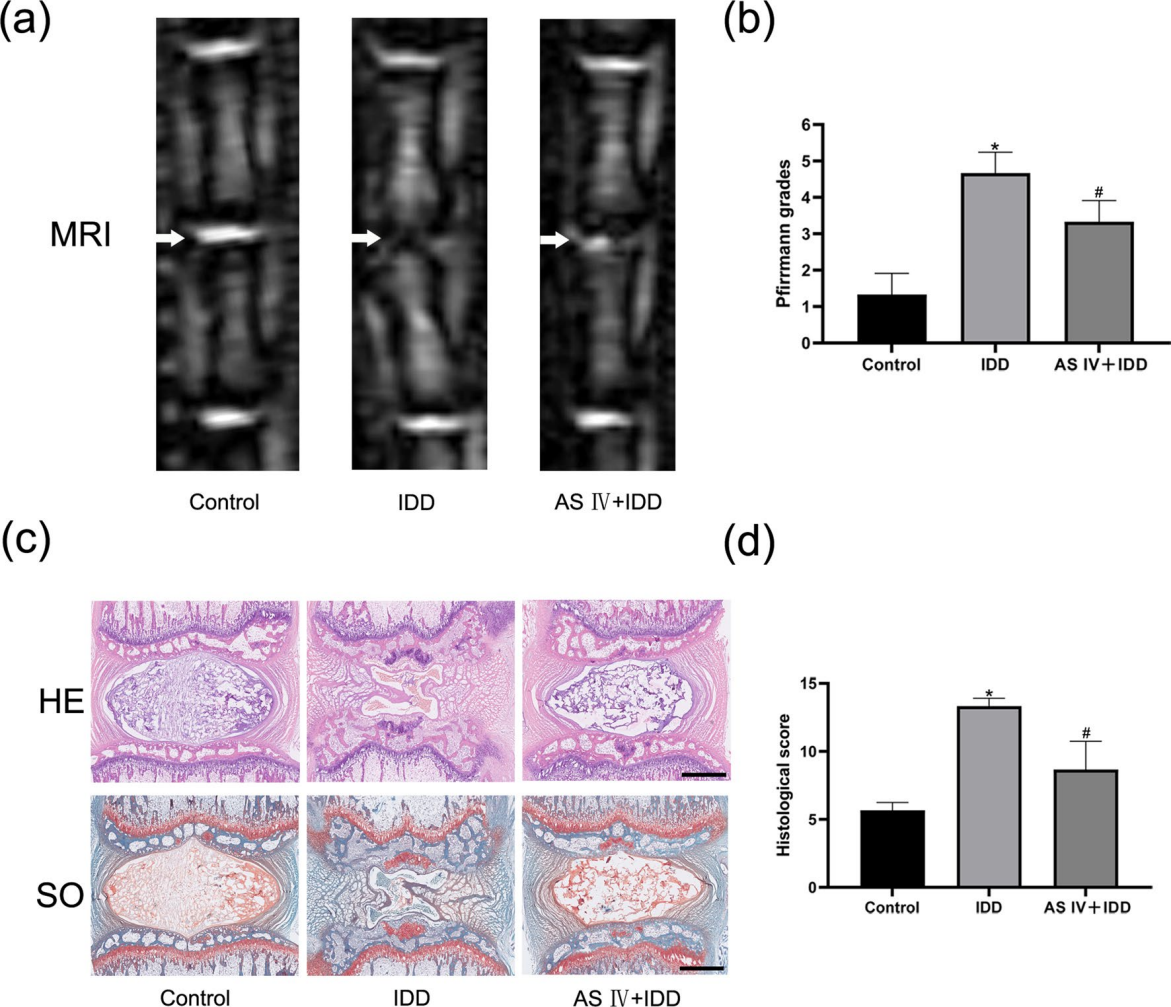
AS IV alleviates IDD in vivo. **a** The tail IVDs of rats were assessed by MRI. **b** Quantitative analysis of IDD using the Pfirrmann MRI grading system. **c** H&E and SO staining of the rat IVDs. Scale bar: 1 mm. **d** The histological scores of the rat IVDs. Data are expressed as means ± SD. *P < 0.05 versus control group. ^#^*P* < 0.05 versus IDD group

To further evaluate the anti-inflammatory properties of AS IV in IDD in vivo, we established a rat tail disc degeneration model induced by a needle puncture procedure. The degree of IDD in rats was assessed by MRI and quantified using the Pfirrmann MRI grading score. Our results showed that the disc structure was uniform in the control group, with high signal intensity and normal disc height (Fig. 5a). However, in the IDD group, the disc structure was not homogeneous, with lower signal intensity. The AS IV-treated group demonstrated a higher T2-weighted signal intensity compared to the untreated IDD group. Furthermore, the Pfirrmann MRI grading score (indicating the degree of IDD) of the AS IV + IDD group was significantly lower than that of the IDD group at 8-week time point (Fig. 5b).

H&E staining showed that at 8 weeks, the NP structure had almost disappeared in the IDD group (Fig. 5c). However, this structure was retained and the morphology of NP tissue in the IVD was still clear in the AS IV-treated IDD group. Furthermore, SO staining, a stain that detects proteoglycans and glycosaminoglycans, showed superior structural and ECM preservation of NP tissue in the AS-IV-treated group compared to the untreated IDD group (Fig. 5c). Histological scoring also confirmed these observations (Fig. 5d). Therefore, our results indicate that AS IV effectively alleviates the progression of IDD in vivo.

## Discussion

Numerous patients are affected by IDD, the condition that leads to low back pain and related complications, seriously damaging the labour force and bringing a heavy economic burden to the society. Current treatments for IDD only temporarily relieve pain, failing to eradicate the causes, and resulting in the series of sequelae. The main reason for this lack of treatment is the fact that the pathogenesis of IDD is not completely clear. Even though an increasing number of scientists is working in this field, the treatment for IDD has not been greatly improved. To identify safer and more effective treatments for IDD, the latest studies focused on the cellular and molecular (pathological) mechanisms related to IDD [32, 33]. It is widely accepted that inflammation plays an important role in the occurrence and aggravation of IDD [34]. Furthermore, the aberrant expression of IL-1β in degenerated IVD, as well as its strong pro-inflammatory properties, have been confirmed by many studies. Here, to induce IDD in vitro, we treated cells with IL-1β, similar to previous studies [35–37]. The normal physiological function of IVD is characterized by ECM homeostasis in NP. The main components of ECM are type II collagen and aggrecan, which can be degraded by several matrix degrading enzymes. A higher rate of ECM degradation (compared to ECM synthesis) leads to IDD. In addition, excessive apoptosis of NPCs reduces the number of live cells, further contributing to decreased NP ECM content and the development of IDD. This progressive degradation of NP ECM and excessive apoptosis of NPCs are important pathological hallmarks of IDD [38, 39]. It has been shown that IL-1β decreases the expression of aggrecan and type II collagen and increases the expression of ADAMTS-4 and MMP-13, which is consistent with our findings. Since IL-1β induces NPC apoptosis, the inhibition of IL-1β-induced NP ECM degradation and NPC apoptosis would be an effective treatment of IDD.

Recently, several Chinese medicinal plants have been shown to have therapeutic properties for the treatment of numerous diseases [40–42]. AS IV, a natural saponin purified from the traditional Chinese herb *Astragalus membranaceus*, has the molecular formula C_41_H_68_O_14_ and numerous pharmacological properties, while its anti-inflammatory properties have been studied the most [17, 18]. Previous studies have shown that AS IV can alleviate chondrocyte degeneration by inhibiting inflammatory responses [23, 24]. Since NP and articular cartilage have similar histocytological composition and biomechanical function [43], we hypothesized that AS IV could be effective for the treatment of IDD. In this study, to mimic IDD in vitro, we stimulated NPCs with IL-1β and then treated these cells with AS IV. RT-qPCR and Western blotting results demonstrated that AS IV markedly reduced the levels of the inflammatory mediators COX-2 and iNOS induced by IL-1β stimulation, suggesting that AS IV effectively inhibited inflammatory responses in vitro. We also evaluated the effect of AS IV on NPC apoptosis. Compared with the IL-1β group, the expression of pro-apoptotic genes (cleaved-caspase-3 and Bax) was downregulated, while the expression of anti-apoptotic molecule (Bcl-2) was upregulated in the AS IV treatment group. The results of flow cytometry further confirmed that AS IV could alleviate NPC apoptosis induced by IL-1β. Next, we evaluated the effect of AS IV on NP ECM degradation. We examined the protein expression of aggrecan and type II collagen, major ECM components, and of the ECM catabolic factors ADAMTS-4 and MMP-13, and showed that AS IV treatment alleviated ECM degradation. Immunofluorescence results further confirmed the protective effect of AS IV on the ECM of NPCs. Collectively, these results indicated that AS IV alleviated NPC apoptosis and ECM degradation.

It has been reported that the NF-κB pathway is an important mediator of the destructive effects induced by IL-1β [44, 45]. Furthermore, NF-κB p65 binding sites have been identified in the promoter regions of several MMP genes [46]. Therefore, we investigated whether AS IV protects NPCs by inhibiting NF-κB signalling. Under normal conditions, NF-κB is located in the cytoplasm as a homo- or heterodimer, forming an inactive complex with the inhibitor kappa B (IκB). Upon IL-1β stimulation, IκBα is phosphorylated, leading to the degradation of IκBα protein and nuclear translocation of free NF-κB. [47] Several studies have verified that the activation of NF-κB signalling pathway is implicated in NPC apoptosis and ECM degeneration [48–51], making it an important contributor to the pathogenesis of IDD. Furthermore, the inhibition of NF-κB signalling pathway has been shown to significantly suppress the progression of IDD, indicating that it is an effective therapeutic target for the treatment of IDD. Our results showed that compared with the IL-1β group, AS IV treatment significantly reduced the cytoplasmic localization of p-IκB, inhibited the degradation of IκB, and decreased NF-κB p65 nuclear translocation in response to IL-1β stimulation. These results indicate that the inhibition of NF-κB signalling correlates with the protective effects of AS IV on NPCs.

To verify whether AS IV is effective for the treatment of IDD in vivo, we utilized a rat needle puncture model of IDD, the model commonly used to study the molecular mechanisms of IDD. MRI results showed that IVDs in the AS-IV-treated group had a higher T2-weighted signal intensity than that in the saline control group. H&E and SO staining confirmed that the AS IV treatment group had a better tissue organization and ECM components than the saline-treated group. The Pfirrmann and histological scores further corroborated that the intragastrical injection of AS IV alleviated IDD.

Here, in this study, we demonstrated for the first time that AS IV has a protective effect on nucleus pulposus cells both in vitro and in vivo, and this effect was due to the inhibition of the NF-κB pathway. However, we only explored the involvement of the NF-κB pathway, while other pathways were not evaluated. In our future experiments, we will evaluate the protective properties of AS IV using the ex vivo compression model, which is considered to be more representative of IDD.

## Conclusion

Here, we demonstrated that AS IV alleviated IL-1β-induced inflammatory responses, apoptosis, and ECM degeneration in human NPCs. Mechanistically, AS IV effectively inhibited the activation of the NF-κB signalling pathway, suggesting that the protective effect of AS IV includes the suppression of NF-κB activation in human NPCs. Furthermore, our findings showed that AS IV can effectively alleviate puncture-induced IDD in vivo, indicating that AS IV has a therapeutic potential as a treatment for IDD.

## Data Availability

The data that support the findings of this study are available from the corresponding author on reasonable request.

## Abbreviations

IDD: Intervertebral disc degeneration
AS IV: Astragaloside IV
NP: Nucleus pulposus
p-IκBα: Phosphorylated inhibitor of kappa B-alpha
IVD: Intervertebral disc
NPCs: NP cells
ECM: Extracellular matrix
CCK-8: Cell counting kit 8
MRI: Magnetic resonance imaging
Col II: Type II collagen
HE: Haematoxylin and eosin
SO: Safranin O-fast green
